# DNA Methylation Pattern in Overweight Women under an Energy-Restricted Diet Supplemented with Fish Oil

**DOI:** 10.1155/2014/675021

**Published:** 2014-01-22

**Authors:** Cátia Lira do Amaral, Fermín I. Milagro, Rui Curi, J. Alfredo Martínez

**Affiliations:** ^1^Department of Nutrition, Food Sciences and Physiology, University of Navarra, C/Irunlarrea 1, 31008 Pamplona, Spain; ^2^Department of Physiology and Biophysics, Institute of Biomedical Sciences, University of São Paulo, Avenue Professor Lineu Prestes, 1524, 05508-900 São Paulo, SP, Brazil; ^3^CIBER Fisiopatología de la Obesidad y la Nutrición (CIBERobn), Instituto de Salud Carlos III, 28029 Madrid, Spain

## Abstract

Dietary factors modulate gene expression and are able to alter epigenetic signatures in peripheral blood mononuclear cells (PBMC). However, there are limited studies about the effects of omega-3 polyunsaturated fatty acids (*n*-3 PUFA) on the epigenetic mechanisms that regulate gene expression. This research investigates the effects of *n*-3-rich fish oil supplementation on DNA methylation profile of several genes whose expression has been reported to be downregulated by *n*-3 PUFA in PBMC: *CD36*, *FFAR3*, *CD14*, *PDK4*, and *FADS1*. Young overweight women were supplemented with fish oil or control in a randomized 8-week intervention trial following a balanced diet with 30% energy restriction. Fatty acid receptor *CD36* decreased DNA methylation at CpG +477 due to energy restriction. Hypocaloric diet-induced weight loss also reduced the methylation percentages of CpG sites located in *CD14*, *PDK4*, and *FADS1*. The methylation patterns of these genes were only slightly affected by the fish oil supplementation, being the most relevant to the attenuation of the weight loss-induced decrease in *CD36* methylation after adjusting by baseline body weight. These results suggest that the *n*-3 PUFA-induced changes in the expression of these genes in PBMC are not mediated by DNA methylation, although other epigenetic mechanisms cannot be discarded.

## 1. Introduction

Several environmental factors, including dietary intake of specific nutrients, germ-line genetic variations, stochastic events, and inheritance systems, may change epigenetic marks and affect gene expression [1, 2]. The most important environmental factors associated with obesity onset are sedentary lifestyles and unbalanced diets, such as the intake of energy-dense foods [3]. The change of dietary habits and physical activity patterns are the main strategy for managing obesity and the associated disorders. Fatty fishes are rich in long chain fatty acids, especially omega-3 polyunsaturated fatty acids (*n*-3 PUFA), as exemplified by salmon and trout [4]. Particularly, eicosapentaenoic acid (EPA; 20:5*n*-3) and docosahexaenoic acid (DHA; 22:6*n*-3) are the *n*-3 PUFA occurring in fish oil that have been more comprehensively studied due to their health improving effects [5]. For instance, the consumption of an overall healthy diet including fatty fish at least twice a week is a recommendation for reducing the risk of developing cardiovascular diseases [6]. Furthermore, *n*-3 PUFA have also been proposed to reduce proinflammatory cytokine production and decrease obesity-induced insulin resistance [7]. Although epidemiological associations between *n*-3 PUFA intake and obesity are inconclusive, some cross-sectional studies have demonstrated inverse relationships between them [8].

Consumption of *n*-3 PUFA affects the expression of many genes in various cell types and tissues, including liver [9], adipose tissue [10], and blood white cells [11, 12]. In peripheral blood mononuclear cells (PBMC), *n*-3 PUFA supplementation has been reported to downregulate the expression of genes related to fatty acid transport, such as fatty acid desaturase 1 (*FADS1*) and fatty acid receptor 3 (*FFAR3*), in insulin resistant subjects [11] as well as other genes such as CD36 molecule (*CD36*), the cluster of differentiation 14 (*CD14*), and pyruvate dehydrogenase kinase 4 (*PDK4*) in healthy elderly subjects [12]. However, few studies have examined the effects of PUFA supplementation on DNA methylation, a key epigenetic mechanism for gene expression regulation. *In vitro*, EPA treatment reduced the demethylation of one CpG at the promoter of CCAAT/enhancer-binding protein *δ* (*C/EBP*δ**) in U937 leukemia cells [13]. In rodents, *n*-3 PUFA supplementation during pregnancy and/or lactation increased liver *FADS2 *DNA methylation in the offspring [14]. *FADS2 *methylation was increased in the liver of dams exposed to an *n*-3 PUFA-deficient diet during pregnancy followed by postnatal supplementation diet containing *n*-3 PUFA from flaxseed oil [15]. On the other hand, *n*-3 PUFA supplementation during pregnancy increased global DNA methylation levels in rats supplemented with folic acid in the absence of vitamin B12 [16]. However, other authors analyzed the methylation pattern of genes in mouse epididymal fat (leptin, leptin receptor, and proopiomelanocortin) and did not find effects as a result of fish oil supplementation [17]. Up to date, there is no report about the effects of *n*-3 PUFA supplementation on DNA methylation in adult humans.

Hence, the aim of the present study was to test the hypothesis whether fish oil supplementation within an energy-restricted dietary treatment affects the DNA methylation pattern of genes that are downregulated by *n*-3 PUFA supplementation in PBMC, such as the fatty acid receptors *CD36* and *FFAR3*, the enzymes *FADS1* and *PDK4*, and the surface antigen *CD14*.

## 2. Methods

### 2.1. Trial Design and Participants

The primary study was based on a randomized 8-week intervention trial study of four isocaloric diets, designed to investigate the specific effects of fish consumption or fish oil supplementation on weight loss in young overweight adults (trial registration: ClinicalTrials.gov NCT00315770) [18]. In this clinical trial, a total of 324 overweight individuals were included (140 from Iceland, 120 from Spain, and 64 from Ireland) and randomly assigned to four diets groups (control, cod, salmon, and fish oil capsules) [18]. Detailed description of the protocol, participant recruitment and enrolment, and inclusion and exclusion criteria are described elsewhere [18, 19]. Briefly, the initial inclusion criteria were body mass index (BMI) 27.5–32.5 kg/m^2^, age between 20 and 40 years old, and waist circumference ≥94 cm and ≥80 cm for men and women, respectively. Exclusion criteria were weight change due to weight-loss diet within 3 months before the start of the study, use of supplements containing *n*-3 fatty acids, calcium, or vitamin-D during the last 3 months, drug treatments of diabetes mellitus, hypertension, or hyperlipidemia, and women's pregnancy or lactation.

**Figure 1:**
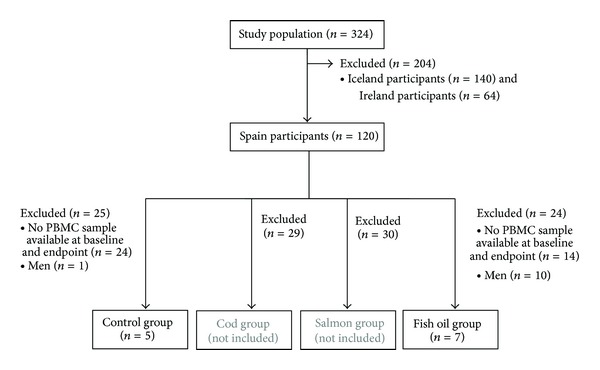
Flow chart of the participants in the study.

As the objective of the paper is to examine the effects of fish oil supplementation on the DNA methylation pattern of genes that are downregulated by *n*-3 PUFA supplementation in PBMC, we only analyzed Spanish subjects from two of the intervention groups: control and fish oil (Figure 1). A selection of the individuals of these two groups was performed, taking into account their age, biochemical, and anthropometric characteristics. The reasons for this selection were that (i) it was necessary to match the individuals according to their age, life style, biochemical, and anthropometric characteristics in order to diminish heterogeneity and to avoid confounding environmental factors and (ii) the high cost of the epigenetic determinations implied that they could not be applied to a large number of patients. In a first moment, two more subjects from each group had been selected but, unfortunately, the quality of their DNA samples was under the quality standards of the procedure and could not be included in the analysis. Because we aimed to evaluate DNA methylation changes in response to fish oil supplementation, we selected participants with PBMC samples available at baseline and endpoint. Furthermore, due to the low number of men in the intervention groups, men were excluded. So, the DNA methylation study was conducted in 12 young overweight/obese women of similar characteristics that belonged to the two following groups: control (*n* = 5) and fish oil (*n* = 7). To reduce variability, subjects belonging to the cod and salmon groups were not analyzed because if changes in DNA methylation were observed in these groups, it would be impossible to find the responsible agents due to the big differences in macronutrients and micronutrients between both fish species. The study was approved by the Ethical Committee of the University of Navarra and followed the Helsinki guidelines. All participants gave their written consent after being informed of the nature purpose and possible risks of the study. This study was performed at the University of Navarra in Pamplona, Spain, and recruitment was undertaken during 2004 and 2005.

### 2.2. Dietary Intervention

Overweight young women were instructed to follow a diet with 30 percent fewer calories than the estimated energy expenditure (approximately 600 kcal/day), for eight consecutive weeks. As described earlier [18], basal metabolic rate was estimated by applying Harris-Benedict equations and a correction factor due to the overweight status of the subjects [20, 21]. To estimate total energy expenditure, the physical activity level was set to 1.3, as a relatively low physical activity level was reported by all subjects [18]. PAL was estimated according to the Nordic Nutrition Recommendations, 2004 [22]. The questionnaire asked about the occupation, sleeping hours, and additional activities at work and during the rest of the day. The physical activity questionnaire included representative values expressed as multiples of Resting Energy Expenditure (REE). Average daily exercise was calculated taking into account the intensity level and time spent on each activity. Finally, activities were quantified with respect to the Resting Energy Expenditure (1.0). The subjects under weight loss treatment were randomly single-blindedly assigned to control group (6 placebo capsules/day) or fish oil group (6 capsules/day). Both capsules were from Loders Croklaan (Lipid Nutrition), Wormerveer, the Netherlands, encapsulated by Banner Pharmacaps, Tilburg, the Netherlands. Participants from both control and fish oil groups were instructed not to eat seafood because it could complicate the analysis that would be translated into unknown variability. So, seafood was simply stopped for the intervention period. Each subject received a detailed meal plan to follow, as well as recipe booklets and instructions, so as to minimize differences between diets in terms of sources of fat, fruit and vegetable consumption, and meal frequency. Diets were similar in total fat (30% of total energy), carbohydrate (50% of total energy), protein (20% of total energy), and dietary fiber (20–25 g) [18, 19]. Amounts of long chain *n*-3 fatty acid intake were <260 mg/day for the control and >1300 mg/day for the fish oil groups, as elsewhere described [23].

### 2.3. Anthropometric and Metabolic Measurements

Anthropometric parameters measurements and blood samples collections were carried out at baseline and after supplementation period (endpoint), as previously described [24]. Plasma levels of glucose, total cholesterol, and triacylglycerols were measured by specific colorimetric assays (Horiba ABX Diagnostics, Montpellier, France) using an automatic system (COBAS MIRA, Roche, Basel, Switzerland), whereas circulating insulin levels were determined by ELISA (Mercodia, AB, Uppsala, Sweden). The homeostasis model assessment index (HOMA-IR) was calculated according to Matthews et al. [25] in order to assess insulin resistance.

### 2.4. PBMC Isolation, DNA Extraction, and Bisulfite Conversion

PBMC were isolated by differential centrifugation using Polymorphprep (Axis Shield PoC AS, Oslo, Norway), as previously described [26], and stored at −80°C. DNA was extracted using AllPrep DNA/RNA Mini Kit (Qiagen, Hilden, Germany). DNA concentration was quantified with Quant-iT PicoGreen dsDNA Assay Kit (Invitrogen, Carlsbad, CA, USA) and genomic DNA (2 *μ*g) was bisulfite-converted with the EpiTect Bisulfite Kit (Qiagen, Valencia, CA, USA).

### 2.5. Methylation Profile Determined by MALDI-TOF Mass Spectrometry

The DNA methylation profiles of five genes were analyzed in PBMC: *CD36*, *CD14*, *FADS1*, *PDK4,* and *FFAR3*. The genomic sequences studied are shown in Table 1S in supplementary materials available online at http://dx.doi.org/10.1155/2014/675021.

Firstly, a region covering 3 kb (2,000 base pairs upstream to 1,000 base pairs downstream from the transcriptional start site) was chosen from Gene Bank (http://www.ncbi.nlm.nih.gov/gene) for each gene. CpG islands were identified using MethPrimer software (http://www.urogene.org/methprimer/) [27]. Whereas three genes showed CpG islands (*CD14*, *PDK4*, and *FADS1*), neither *FFAR3* nor *CD36 *showed these CpG dinucleotide-rich regions. Predicted transcription factor binding sites were identified with AliBaba2 software (http://www.gene-regulation.com/pub/programs/alibaba2/index.html) with a homology of 75%. Then, a region of 300 to 500 bp in a CpG island and/or rich in predicted transcription factors was chosen for each gene (Table 1). The methylation profile of these genes was determined by Sequenom's MassARRAY EpiTyper technology (Sequenom, San Diego, CA, USA), which relies on base-specific cleavage followed by MALDI-TOF mass spectrometry as previously described [28]. The primers used are reported in Table 1. DNA methylation values of some CpG sites could not be measured independently. This is the case of nearby CpG sites and CpG sites included in similar chemical fragments after the enzymatic breakdown. So, they were clustered together and were considered as an independent CpG site when analyzed.

### 2.6. Statistical Analysis

The data are presented as the mean ± SD and were analyzed using SPSS 15.0 for Windows (SPSS, Chicago, IL, USA). A general linear model with repeated measures was conducted to assess the impact of oral supplementation (control or fish oil) on DNA methylation of *CD36*, *FFAR3, CD14*, *PDK4*, and *FADS1 *through the weight loss treatment (baseline and endpoint). For CpG site +477 from CD36 gene, we hypothesized that the differences in initial body weight may affect statistical analysis. In order to control the effect of the body weight, statistical analyses were conducted with baseline body weight as a covariable. The significance level was set at *P* < 0.05.

## 3. Results

### 3.1. Baseline DNA Methylation Profile

**Figure 2:**
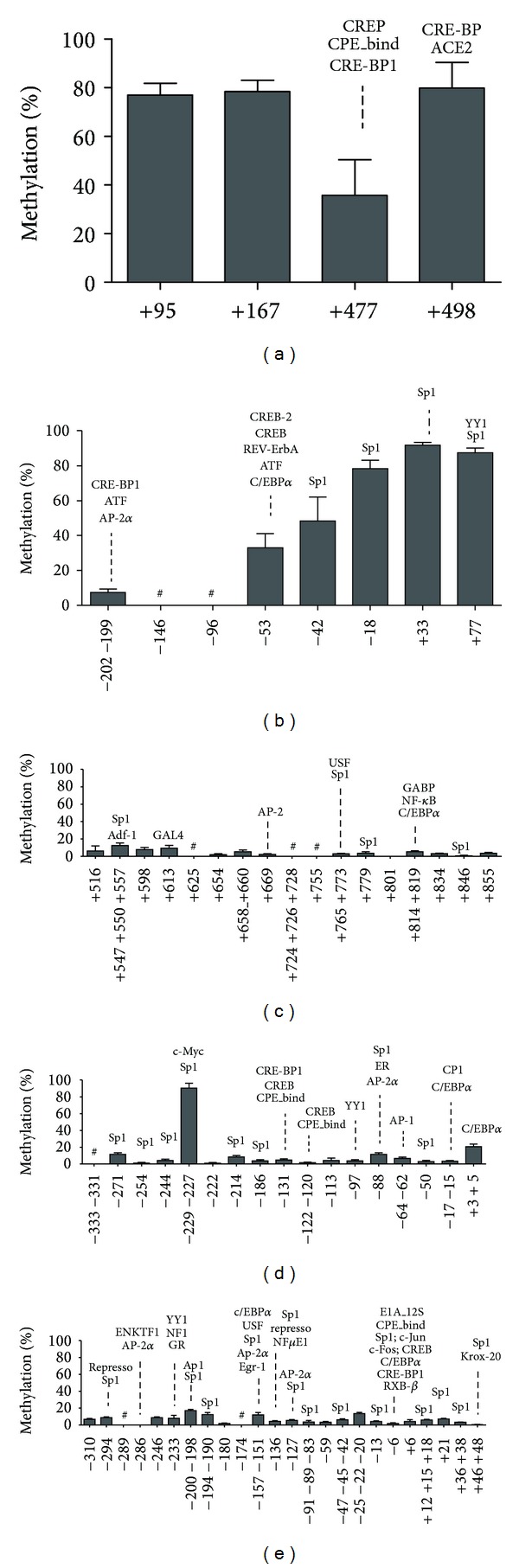
Baseline DNA methylation profile of the genes (a) *CD36*, (b) *FFAR3*, (c) *CD14*, (d) *PDK4*, and (e) *FADS1* as determined in PBMC from overweight young women before weight loss treatment. The bars represent methylation levels of each CpG and are numbered according to the transcription start site. The predicted binding sites for transcription factors that are associated with the CpGs (identified by AliBaba software with a homology of 75%) are also indicated. Data are mean ± SD, *n* = 12. ^#^Not analyzed.

Recruitment was undertaken during 2004 and 2005. A total of 12 women were selected for the present study (Figure 1). Different patterns of methylation were found in the genes studied in PBMC (Figure 2). Low methylation levels (less than 25% [29]) were observed in the genes that encode the superficial molecule *CD14* and the metabolic enzymes* PDK4* and* FADS1*, although one CpG of *PDK4 *(−229−227) was highly methylated (more than 75% [29]). *CD36* studied region was highly methylated, whereas *FFAR3* showed two distinct regions: a highly methylated region close to the transcription start site (CpG sites −18, +33, and +77) and a low-methylated region between CpGs −53 and −202.

### 3.2. Weight Loss Treatment and Fish Oil Supplementation Effects on DNA Methylation Profile

Both experimental groups (control and fish oil) responded similarly to the weight loss treatment (Table 2), showing similar reductions in body weight, BMI, and serum leptin levels (*P* < 0.05) but no effects on other metabolic parameters.

**Figure 3:**
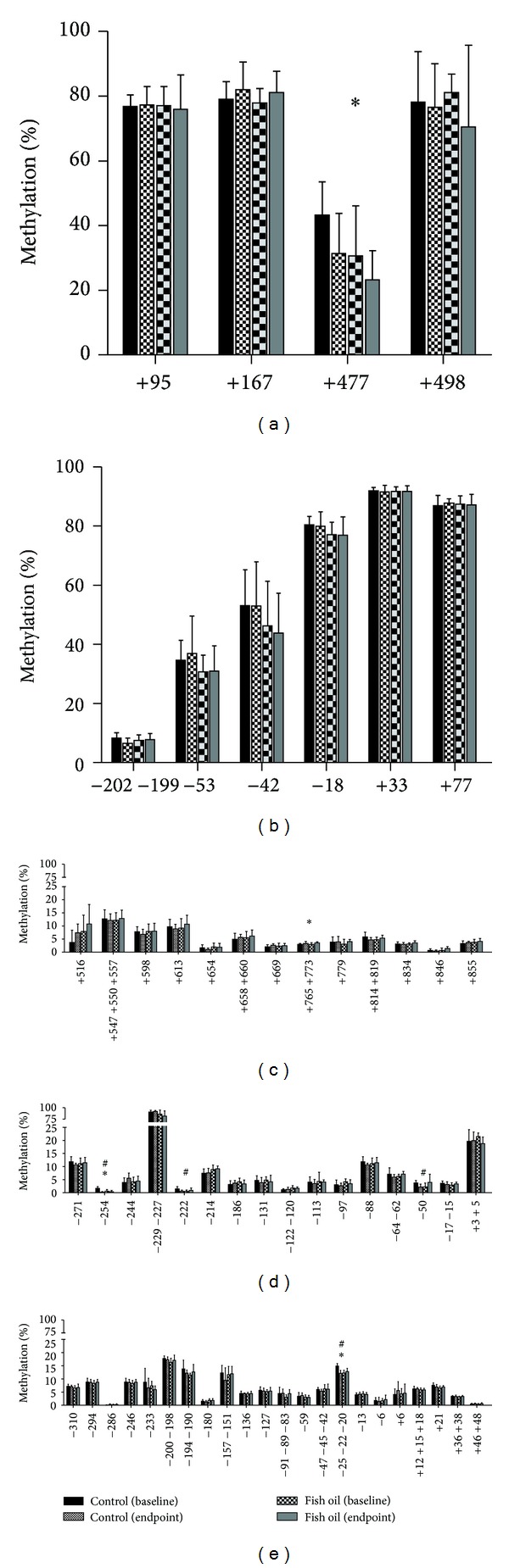
Methylation of the CpGs located in the studied regions of the genes (a) *CD36*, (b) *FFAR3*, (c) *CD14*, (d) *PDK4*, and (e) *FADS1* in PBMC from control and fish oil-supplemented women during the 8-week weight loss treatment. Data represent mean ± SD (control *n* = 5; fish oil *n* = 7). DNA methylation was determined at baseline and endpoint samples by MassARRAY EpiTyper in triplicate. A general linear model with repeated measures was used to assess the impact of oral supplementation on DNA methylation (between subjects) across the weight loss treatment (within subjects). *P < 0.05, differences due to weight loss (baseline versus endpoint); ^#^ *P* < 0.05, interaction between weight loss effect and oral supplementation.

Hypocaloric diet-induced weight loss affected DNA methylation in the *CD36* gene, reducing the percentage of methylation of CpG +477 (Figure 3(a)). This CpG lies in a putative binding site for the transcription factors CREB and CRE-BP1 (Figure 2(a), CpG +477). However, fish oil supplementation did not alter the DNA methylation pattern of *CD36* gene, with reductions of 11.8% in the methylation of CpG +477 in the control group and 7.3% in the fish oil group. In order to control the effect of body weight, methylation status was adjusted to baseline body weight. In this case, the methylation of CpG +477 was lower (*P* < 0.05) in the fish oil group (6.7%) than in the control group (12.7%).

Energy restriction also affected DNA methylation in *CD14* (Figure 3(c)) but only with a very slight increase of 0.3% and 0.6% at the CpGs +765+773 in the control and the fish oil groups, respectively. These CpGs are included in a putative binding site for the transcription factors USF and Sp1 (Figure 2(c)).

Statistically significant (although small) changes were also induced by the hypocaloric diet-induced weight loss in the promoters of *PDK4* and *FADS1* genes (CpGs −254 and −25−22−20, resp., in Figures 3(d) and 3(e)). Furthermore, the only CpGs that showed significant methylation differences between the control and the fish oil groups are in these genes. Thus, the methylation percentage of *PDK4* CpG sites −222 and −50 and *FADS1* CpG −25−22−20 were reduced in the control group and increased with fish oil, whereas the reduction observed in *PDK4* CpG −254 in the controls was greater than in the fish oil group. Interestingly, *PDK4* CpGs −254 and −50 lay in putative binding sites for the transcription factor Sp1 (Figure 2(d)). Nevertheless, although statistically significant, these differences were so small that they probably do not influence gene expression.

## 4. Discussion

Although there is strong evidence of the modulatory effects of *n*-3 PUFA on gene expression in several cell types in humans, it is not known if they could act on gene expression by altering the epigenetic mechanism of DNA methylation. Herein, we sought to determine the methylation profiles of *CD36*, *FFAR3*, *CD14*, *PDK4*, and *FADS1* in PBMC and whether this methylation is affected by a weight loss nutritional intervention (energy-restriction) with or without fish oil supplementation in young overweight women. As expected, phenotypic changes due to energy restriction led to a reduction in body weight. In humans, energy-restricted diets modify DNA methylation in PBMC [28]. Our results corroborate these findings, suggesting that weight loss treatment induces small changes in the methylation profile of specific genes from blood cells (i.e., *CD36*, *CD14*, *PDK4*, and *FADS1*).

*CD14*, *PDK4*, and *FADS1* have low methylation percentages (<25%) in PBMC before starting nutritional intervention (Figure 2). Similar results have been reported for *PDK4* (<10%) in human skeletal muscle [30] and for *CD14* and *FADS1* in PBMC [31] and murine aorta [32], respectively. On the other hand, *CD36* and *FFAR3* have been found to be highly methylated in PBMC. Whereas the methylation of *CD36* has not been previously described, a hypomethylated region close to *FFAR3* has been reported in the adult hematopoietic compartment [33].

The five genes studied have been reported to be downregulated in PBMC from fish oil-supplemented subjects [11, 12]. Although few studies (all of them in animal models) have analyzed the effect of fish oil on DNA methylation, it has been observed that the epigenetic effects of *n*-3 PUFA depend on the gene and the tissue and are far more relevant during pregnancy and lactation than during adult life [14–17]. In the current study, very small methylation differences have been found between the control and the fish oil groups. Importantly, fish oil supplementation did not potentiate the 8-week hypocaloric diet-induced weight loss in our study. Although it has been described that fish oil supplementation could improve weight loss in men with caloric restriction [18], other studies have not found an improvement of weight loss in postmenopausal women with type 2 diabetes [34] or hyperinsulinemia [35]. This finding is important for approaching the discussion of the methylation results because the changes observed in the *n*-3 PUFA-supplemented group cannot be attributed to shifts in anthropometric or biochemical parameters with the intervention.

The most relevant effect due to *n*-3 PUFA supplementation was found in *CD36* CpG +477 (after adjusting for baseline body weight). *CD36* encodes a membrane glycoprotein that belongs to the class B scavenger receptor family that may play an important role in lipid metabolism in humans and may be involved in obesity-related complications [36]. In macrophages, it is responsible for binding and internalization of oxidized LDL while in metabolically active tissues, especially in adipocytes, heart and skeletal muscle, *CD36* mediates the uptake of long-chain fatty acids across the plasma membrane [36]. In macrophages and monocytes, CD36 promotes lipid uptake leading to activation of *PPAR*γ** transcriptional pathways and may also promote inflammatory response and phagocytosis [37]. Thus, THP-1 macrophages treated with oleic acid, linoleic acid, EPA, and DHA showed increased *CD36* mRNA expression [38]. Similarly, the expression of *CD36* was increased in PBMC after oral acute intake of MUFA-rich virgin olive oil [39] and in monocytes after 24 h lipid infusion [40] in humans. On the other hand, the expression of *CD36* was downregulated in PBMC due to a long-term supplementation with EPA/DHA [12]. These results indicate that the type of fatty acids (PUFA or MUFA) regulates *CD36* expression in a different manner depending also on the time (acute *versus* chronic). In our 8-week long study, the hypocaloric diet-induced weight loss decreased *CD36* gene DNA methylation (in CpG +477) with less intensity in the fish oil-supplemented group than in the controls. However, the small decrease observed does not, probably, influence the regulation of *CD36* gene expression.

An epigenetic change may influence *CD36* gene expression in blood cells and may contribute to modify inflammatory monocyte/macrophage activation during weight loss treatment. This notion is supported by the fact that PBMC from obese women have a proinflammatory state, with increased expression in PBMC of proinflammatory genes like tumor necrosis factor-*α* (*TNF-*α**), interleukin-6 (*IL-6*), migration inhibitor factor (*MIF*), and matrix metalloproteinase-9 (*MMP9*) and an increase in NF-*κ*B binding to DNA [41]. *CD36* CpG +477 lies in a recognition motif for CRE-BP1 and CREB, members of the leucine zipper family of DNA binding proteins that bind to cAMP-response elements (CRE) [42]. Both transcription factors (CREB and CRE-BP1) also interact with CREB binding protein (CBP)/p300 associating factor, which, as CRE-BP1, is a known HAT or histone acetyltransferase [43, 44] and may promote chromatin modifications by inducing histone acetylation. If transcription factor-DNA binding affinity is affected by cytosine methylation, the HAT activity might be downregulated and histone deacetylation increased, which could justify the underexpression of the target genes, as described in PBMC from subjects supplemented with fish oil [12]. Besides the small number of participants included in the present study, we found a clear effect of weight loss treatment on the methylation percentage of many CpG sites, especially in the promoter region of *CD36*. Our translational research demonstrates that dietary energy-restriction results in epigenetic adaptations in PBMC cells. However, as only 12 subjects were analyzed, the results could not be extrapolated to the whole population and should be validated in larger and more complex populations. In any case, the results are of very high value because the individuals have been carefully selected from a big population and matched trying to minimize other confounding factors apart from the dietary intervention (fish oil capsules or not).

The small differences (<2%) observed in DNA methylation between the control and the fish oil groups in CpGs located in* PDK4* and *FADS1*, although statistically significant, probably do not affect the expression of the genes. Both genes have been previously reported to be regulated by DNA methylation: physical exercise immediately decreased *PDK4* methylation in skeletal muscle [45], whereas subjects with type 2 diabetes presented higher *PDK4* expression in skeletal muscle accompanied by a tendency to reduce promoter DNA methylation when compared with normal glucose tolerance subjects [30]. On the other hand, *FADS1* expression was regulated by DNA methylation in a gastric cancer cell line as demonstrated by treating the cells with the demethylating agent 5-aza-2′-deoxycytidine, which increased by 23-fold *FADS1* mRNA expression [46]. A recent study did not find modifications in *FADS1* methylation in offspring aorta as a result of changes in the lipid composition of the maternal diet [32]. Considering the transcription factors associated with the CpG sites affected, Sp1 is an ubiquitously expressed transcription factor that participates in the overexpression of *CD14* during monocytic cell differentiation by interacting with another protein, MEF2D [47]. However, the small methylation changes observed in this study probably do not affect the binding properties of Sp1 in the studied genes.

In summary, an 8-week weight-loss nutrition intervention not only reduced body weight and circulating leptin in overweight women but also modified DNA methylation of *CD36* in PBMC and, very slightly, of *CD14*, *PDK4*, and* FADS1*. Although fish oil oral supplementation did not potentiate the weight-loss effects of the hypocaloric diet, it modulated DNA methylation, that is, reducing the decrease in DNA methylation of *CD36* CpG +477 (after adjusting by baseline body weight). These results should be interpreted with care because the methylation differences are rather small and restricted to few CpG sites, but it should be highlighted that other nutritional studies, including several ones focused on body weight loss [29, 48, 49], have found DNA methylation changes of similar magnitude.

In any case, to be sure that the beneficial effects of EPA/DHA in obesity, type 2 diabetes, and other metabolic diseases are mediated by changes in the DNA methylation pattern, in addition to PBMC, other tissues (such as adipose depots, liver, or skeletal muscle) should be analyzed. Finally, the changes in DNA methylation should be compared with those in the expression of the corresponding genes in these tissues.

## Supplementary Material

Supplementary Material contain genomic sequences analyzed.

## Figures and Tables

**Table 1:** Primers used for the bisulfite conversion of DNA.

Symbol	Primers	Product size	CpG in region^a^
*CD36 *	L: GGGTTGAGAGTTTGTGTTTTATTTTT R: TTCAAATACAATTACACTTTTTAAAATCAC	479	4
*FFAR3 *	L: GGTAAATTGGATAAATGTTATTTTAGAGA R: CAACAAAAAAACACCAAAATACTCC	385	9 (7)
*CD14 *	L: GGAGGGAATTGAATGATATTTTAGG R: AAATCTCCACCTCTACTACAAACACA	429	24 (20)
*PDK4 *	L: TTTTGTTTTGAGTAAGGATTAATGA R: TCCCAAACAAAAAAAATCACTAAAA	400	23
*FADS1 *	L: TTGTAATTTTTAAGGGTTTTTAGGT R: AAACAACTCACAACTAAACTACCAACA	419	37 (35)

^a^Values between parenthesis indicate the number of CpGs covered by Sequenom EpiTyper approach. L: left primer; R: right primer; Chr: chromosome; *CD36*: thrombospondin receptor; *CD14*: cluster of differentiation 14; *FADS1*: fatty acid desaturase 1; *PDK4*: pyruvate dehydrogenase kinase, isozyme 4; *FFAR3*: free fatty acid receptor 3.

**Table 2:** Anthropometric and serum variables from control and fish oil supplemented women before and after the 8-week weight loss treatment.

	Control		Fish oil		Interaction	Weight loss treatment	Oral suppl.
	Baseline	Endpoint	Baseline	Endpoint			
Anthropometric							
Age, years	35.1 ± 5.5	—	32.4 ± 5.0	—			
Height, m	1.65 ± 0.06	—	1.61 ± 0.05	—			
Weight, kg	82.9 ± 2.3	78.7 ± 3.0	78.8 ± 5.2	73.6 ± 5.6	ns.	*P* < 0.05	ns.
BMI, kg/m^2^	30.7 ± 2.0	29.1 ± 2.6	30.5 ± 1.7	28.4 ± 1.8	ns.	*P* < 0.05	ns.
SBP, mmHg	120.6 ± 9.6	121.8 ± 8.3	115.4 ± 9.5	113.1 ± 5.7	ns.	ns.	ns.
DBP, mmHg	67.2 ± 4.1	68.4 ± 2.9	72.1 ± 6.5	68.9 ± 5.6	ns.	ns.	ns.
Serum							
Glucose, mg/dL	86.9 ± 4.6	85.1 ± 3.2	88.1 ± 6.0	90.6 ± 9.7	ns.	ns.	ns.
Insulin, mU/L	8.4 ± 4.0	8.2 ± 4.0	12.4 ± 6.7	8.3 ± 3.3	ns.	ns.	ns.
HOMA index	1.8 ± 0.9	1.7 ± 0.7	2.7 ± 1.5	1.8 ± 0.7	ns.	ns.	ns.
Leptin, *μ*g/mL	37.6 ± 10.7	25.4 ± 8.4	30.6 ± 14.5	18.5 ± 8.0	ns.	*P* < 0.05	ns.
Total cholesterol, mg/dL	214.9 ± 49.0	200.1 ± 44.9	197.4 ± 46.5	185.4 ± 35.1	ns.	ns.	ns.
LDL cholesterol, mg/dL	132.5 ± 30.9	124.8 ± 26.9	114.0 ± 40.6	114.5 ± 29.7	ns.	ns.	ns.
HDL cholesterol, mg/dL	65.2 ± 21.2	58.2 ± 24.7	57.0 ± 11.0	52.7 ± 8.8	ns.	ns.	ns.
Triglycerides, mg/dL	85.9 ± 48.7	85.8 ± 26.1	131.9 ± 75.0	88.1 ± 35.3	ns.	ns.	ns.

Data represent mean ± SD (control *n* = 5; fish oil group *n* = 7). Differences within and between subjects were determined by a general linear model with repeated measures. ns.: not significant; SBP: systolic blood pressure; DBP: diastolic blood pressure; HOMA: homeostasis model assessment; Oral suppl.: effect of the oral supplementation of fish oil.
