# Supplementary material for: DNA Methylation Pattern in Overweight Women under an Energy-Restricted Diet Supplemented with Fish Oil
Source: Biomed Res Int. 2014 Jan 22;2014:675021. doi: 10.1155/2014/675021 (PMC3919118; doi:10.1155/2014/675021)
Supplement: Supplementary file 1 — Supplementary Material contain genomic sequences analyzed. [file 675021.f1.pdf]

**Supplementary table 1S**. Genomic sequences analyzed by Sequenom EpiTyper technology and the CpG dinucleotides highlighted.

| Gene | Genomic sequence |
|---|---|
| *CD36*<br><br>NC_000007 REGION: 80231554..80232032 gene ID: 948, updated on 09-02-2012 | GGGTTGAGAGCCTGTGCCTCATTTCTGAGTTCTCAGCTGCTATGC(+95)**CG**TGGAAATCCTGTTTACTTTC<br>TGCATCTGCTCCTGCAAGACTCTGGAGCCAGTCTTGAGGTCCTACATCTC(+167)**CG**AAAGCAAGCTCTTCT<br>AGAAGTTGGTGAGCAGAATGCTTTTTGTTTAGAAGTGTGGCTATAAATGTCTTTGTAGTTGATGCAC<br>TCTCATCTAGTAAGTAACTATTATTTTTGGGCTCTCTAGAAAGGTAATTATTATCTGATATAATAGT<br>TTAGTCTGTGATGCTTCTTTTAACATATTTGTAAGTTTTAACCAAATGGTTAAAGAAATTTGCTTTT<br>TAACCCTTAAACCTCACATATCCACAAGTCTCTAAATTCCATAGGATGCTATGGATTTCTAGTTGCC<br>TAGTTCATGTCTTTTACTTAGAAAA(+477)**CG**TCAGAAAACCCAAACTTCT(+498)**CG**TGACTTCAAAAAGTGTAAT<br>TGTACCTGAA |
| *FFAR3*<br><br>NC_000019 REGION: 35849221..35849605 gene ID: 2865, updated on 08-16-2012 | GGCAAATTGGATAAATGCCATTCTAGAGAAGCAGACAAAATTCAAGTGAAGAAGGGGAGAGGAAGA(-202)**C**<br>**G**T(-199)**CG**GCTGGGGCCTGCTTAGAGCATCCCAGCTGAGACTGCATGAGGAGGGAGGCA(-146)**CG**CAGTTGTGGA<br>ATTTGTTCCCCTTTTAGCATGCTGACCAGCCCTGGCAA(-96)**CG**GAGCTCAAGGCATCTATGTGCCACTGC<br>TCAACAGTGAGTGA(-53)**CG**TCATGGGCA(-42)**CG**GCCAGGTCTTTATCAGTTCTGC(-18)**CG**GATAAATAGCCAACTG<br>CACTAGGTCTGGAGAGACAGCAAGGTGCTGTG(+33)**CG**GCAGAGCATTTGGGGTCTCAAAGAAGCAGGTGA<br>GCCTGGGCC(+77)**CG**AGGGGCTGGGTGGAGGAGCACCTTGGTGCTTCTCTGCTG |

| | |
|---|---|
| *CD14*<br><br>NC_000005 REGION: 140012391..140012819 (complement)<br>gene ID: 929, updated on 09-06-2012 | +516<br>GGAGGGAACTGAATGACATCCCAGGATTACATAAACTGTCAGAGGCAGC**CG**AAGAGTTCACAAGTGT<br>+547 +550 +557 +598<br>GAAGCCTGGAAGC**CG**G**CG**GGTGC**CG**CTGTGTAGGAAAGAAGCTAAAGCACTTCCAGAGCCTGTC**CG**G<br>+613 +625 +654 +658 +660<br>AGCTCAGAGGTT**CG**GAAGACTTAT**CG**ACCATGGTGAGTGTAGGGTCTTGGGGT**CG**AA**CGCG**TGCCAC<br>+669 +724 +726 +728<br>T**CG**GGAGCCACAGGGGTTGGATGGGGCCTCCTAGACCTCTGCTCTCTCCCCAGGAG**CGCGCG**TCCTG<br>+755 +765 +773 +779 +801<br>CTTGTTGCTGCTGCTGCTGC**CG**CTGGTGCA**CG**TCTCTG**CG**ACCA**CG**CCAGAACCTTGTGAGCTGGA**C**<br>+814 +819 +834 +846 +855<br>**G**ATGAAGATTTC**CG**CTG**CG**TCTGCAACTTCTC**CG**AACCTCAGCC**CG**ACTGGTC**CG**AAGCCTTCCAGT<br><br>GTGTGTCTGCAGTAGAGGTGGAGATCC |
| *PDK4*<br><br>NC_000007 REGION: 95225885..95226284 (complement)<br>gene ID: 5166, updated on 08-16-2012 | -333 -331<br>CCCTGCTCTGAGCAAGGACCAATGAGCA**CGCG**GAGTCCAAACTCTTGGAAACAGTTTGTGGCCAGGA<br>-271 -254 -244 -229<br>GTACTTGACATTGAGACAGCCTC**CG**AGTTGTAAACAAGGG**CG**AGCCTGGG**CG**GGACCCCAGCCCA**CG**<br>-227 -222 -214 -186<br>**CG**ACT**CG**GAGCCC**CG**TCCCAAGAGGCTAATCTTAAGCCCA**CG**TTGCCCCAGATACCTGTTTCTGCTT<br>-131 -122 -120 -113 -97<br>CCTCTTCCCTGTTCTTCCCACCCTTTTTC**CG**TCACAGC**CGCG**GGCAC**CG**GTGCCAGGGCACTC**CG**TG<br>-88 -64 -62 -50<br>GTCAC**CG**TGCCAGGCCATTCTGTGATGAC**CGCG**GCTGGAGGGG**CG**GAGCCCATAGTTCTTTCTCTGA<br>-17 -15 +3 +5<br>TCTGATTGG**CGCG**ACCTGGAGTTCAGGA**CGCG**TTTCCAAGTTCCAGTGACTCCTCCTGTTTGGGA |
| *FADS1*<br><br>NC_000011 REGION: 61584453..61584871 (complement)<br>gene ID: 3992, updated on 08-25-2012 | -310 -294 -289 -286<br>CTGCAACTTTCAAGGGCTCTCAGGCTGCTACTT**CG**GGCAGCACAATTGG**CG**GCA**CG**A**CG**TGGCAAGC<br>-246 -233<br>AGGCAGTAGTTTCCAACCCTGGAGGGTCAG**CG**TCTGGAGACCC**CG**GCCAAGGCATCCACAGCCTAAA<br>-200 -198 -194 -190 -180 -174 -157 -151<br>GATGATGTC**CGCG**AC**CG**CC**CG**GGCAGCCT**CG**TGCA**CG**GAAAAACCTCAACCC**CG**GCCC**CG**CCCACCC<br>-136 -127 -91 -89 -83<br>TTCCTG**CG**GCCACCC**CG**CAGCCCTGGCCCCTCAGTCCATTCACTCCTGCAG**CGCG**GCCC**CG**CACCCA<br>-59 -47 -45 -42 -25 -22 -20 -13<br>GGGCCTGCACTAGAAC**CG**CTGTTCCTAC**CGCG**G**CG**CCCCCTGGGAGCCAA**CG**C**CGCG**ATGCC**CG**CCT<br>-6 +6 +12 +15 +18 +21 +36 +38 +46 +48<br>GA**CG**TCAGGAAGT**CG**AATC**CG**G**CG**G**CG**A**CG**CCTTTAGGGAGCC**CGCG**AGGGGG**CGCG**TGTTGGCAGC<br><br>CCAGCTGTGAGTTGCCC |
